# Supplementary material for: Baseline Characterization of the Gut Microbiota of Field and Colony Populations of Phlebotomus tobbi and Preliminary Assessment of the Anti-Leishmanial Activity of Cultivable Bacteria
Source: Pathogens. 2026 Jun 23;15(7):658. doi: 10.3390/pathogens15070658 (PMC13416292; doi:10.3390/pathogens15070658)
Supplement: Supplementary file 1 [file pathogens-15-00658-s001.zip › pathogens-4357168-File S1/Taxa2ASV_Decomposer/Genus/g__Pantoea/seqs.align.html]

```
seq20           AGTGGGGAATATTGCACAATGGGCGCAAGCCTGATGCAGCCATGCCGCGTGTATGAAGAA
seq21           AGTGGGGAATATTGCACAATGGGCGCAAGCCTGATGCAGCCATGCCGCGTGTATGAAGAA
seq33           AGTGGGGAATATTGCACAATGGGCGCAAGCCTGATGCAGCCATGCCGCGTGTATGAAGAA
seq67           AGTGGGGAATATTGCACAATGGGCGCAAGCCTGATGCAGCCATGCCGCGTGTATGAAGAA
seq35           AGTGGGGAATATTGCACAATGGGCGCAAGCCTGATGCAGCCATGCCGCGTGTATGAAGAA
seq52           AGTGGGGAATATTGCACAATGGGCGCAAGCCTGATGCAGCCATGCCGCGTGTATGAAGAA
seq19           AGTGGGGAATATTGCACAATGGGCGCAAGCCTGATGCAGCCATGCCGCGTGTATGAAGAA
seq40           AGTGGGGAATATTGCACAATGGGCGCAAGCCTGATGCAGCCATGCCGCGTGTATGAAGAA
seq43           AGTGGGGAATATTGCACAATGGGCGCAAGCCTGATGCAGCCATGCCGCGTGTATGAAGAA

seq20           GGCCTTCGGGTTGTAAAGTACTTTCAGCGGGGAGGAAGGCGATGtGGTTAATAACCgCGT
seq21           GGCCTTCGGGTTGTAAAGTACTTTCAGCGGGGAGGAAGGCGATGtGGTTAATAACCgCaT
seq33           GGCCTTCGGGTTGTAAAGTACTTTCAGCGGGGAGGAAGGCGATGcGGTTAATAACCgCGT
seq67           GGCCTTCGGGTTGTAAAGTACTTTCAGCGGGGAGGAAGGCGAcGgGGTTAATAACCctGT
seq35           GGCCTTCGGGTTGTAAAGTACTTTCAGCGGGGAGGAAGGCGAcGgGGTTAATAACCctGT
seq52           GGCCTTCGGGTTGTAAAGTACTTTCAGCGGGGAGGAAGGCGATGgGGTTAATAACCctGT
seq19           GGCCTTCGGGTTGTAAAGTACTTTCAGCGGGGAGGAAGGCGATaAGGTTAATAACCttGT
seq40           GGCCTTCGGGTTGTAAAGTACTTTCAGCGGGGAGGAAGGCGgTGAGGTTAATAACCtCac
seq43           GGCCTTCGGGTTGTAAAGTACTTTCAGCGGGGAGGAAGGCGgTGAGGTTAATAACCtCGc

seq20           CGATTGACGTTACCCGCAGAAGAAGCACCGGCTAACTCCGTGCCAGCAGCCGCGGTAATA
seq21           tGATTGACGTTACCCGCAGAAGAAGCACCGGCTAACTCCGTGCCAGCAGCCGCGGTAATA
seq33           CGATTGACGTTACCCGCAGAAGAAGCACCGGCTAACTCCGTGCCAGCAGCCGCGGTAATA
seq67           CGATTGACGTTACCCGCAGAAGAAGCACCGGCTAACTCCGTGCCAGCAGCCGCGGTAATA
seq35           CGATTGACGTTACCCGCAGAAGAAGCACCGGCTAACTCCGTGCCAGCAGCCGCGGTAATA
seq52           CGATTGACGTTACCCGCAGAAGAAGCACCGGCTAACTCCGTGCCAGCAGCCGCGGTAATA
seq19           CGATTGACGTTACCCGCAGAAGAAGCACCGGCTAACTCCGTGCCAGCAGCCGCGGTAATA
seq40           CGATTGACGTTACCCGCAGAAGAAGCACCGGCTAACTCCGTGCCAGCAGCCGCGGTAATA
seq43           CGATTGACGTTACCCGCAGAAGAAGCACCGGCTAACTCCGTGCCAGCAGCCGCGGTAATA

seq20           CGGAGGGTGCAAGCGTTAATCGGAATTACTGGGCGTAAAGCGCACGCAGGCGGTCTGTTA
seq21           CGGAGGGTGCAAGCGTTAATCGGAATTACTGGGCGTAAAGCGCACGCAGGCGGTCTGTTA
seq33           CGGAGGGTGCAAGCGTTAATCGGAATTACTGGGCGTAAAGCGCACGCAGGCGGTCTGTTA
seq67           CGGAGGGTGCAAGCGTTAATCGGAATTACTGGGCGTAAAGCGCACGCAGGCGGTCTGTTA
seq35           CGGAGGGTGCAAGCGTTAATCGGAATTACTGGGCGTAAAGCGCACGCAGGCGGTCTGTTA
seq52           CGGAGGGTGCAAGCGTTAATCGGAATTACTGGGCGTAAAGCGCACGCAGGCGGTCTGTTA
seq19           CGGAGGGTGCAAGCGTTAATCGGAATTACTGGGCGTAAAGCGCACGCAGGCGGTCTGTTA
seq40           CGGAGGGTGCAAGCGTTAATCGGAATTACTGGGCGTAAAGCGCACGCAGGCGGTCTGTTA
seq43           CGGAGGGTGCAAGCGTTAATCGGAATTACTGGGCGTAAAGCGCACGCAGGCGGTCTGTTA

seq20           AGTCAGATGTGAAATCCCCGGGCTTAACCTGGGAACTGCATTTGAAACTGGCAGGCTTGA
seq21           AGTCAGATGTGAAATCCCCGGGCTTAACCTGGGAACTGCATTTGAAACTGGCAGGCTTGA
seq33           AGTCAGATGTGAAATCCCCGGGCTTAACCTGGGAACTGCATTTGAAACTGGCAGGCTTGA
seq67           AGTCAGATGTGAAATCCCCGGGCTTAACCTGGGAACTGCATTcGAAACTGGCAGGCTaGA
seq35           AGTCAGATGTGAAATCCCCGGGCTTAACCTGGGAACTGCATTTGAAACTGGCAGGCTTGA
seq52           AGTCAGATGTGAAATCCCCGGGCTTAACCTGGGAACTGCATTTGAAACTGGCAGGCTTGA
seq19           AGTCAGATGTGAAATCCCCGGGCTTAACCTGGGAACTGCATTTGAAACTGGCAGGCTTGA
seq40           AGTCAGATGTGAAATCCCCGGGCTTAACCTGGGAACTGCATTTGAAACTGGCAGGCTTGA
seq43           AGTCAGATGTGAAATCCCCGGGCTTAACCTGGGAACTGCATTTGAAACTGGCAGGCTTGA

seq20           GTCTCGTAGAGGGGGGTAGAATTCCAGGTGTAGCGGTGAAATGCGTAGAGATCTGGAGGA
seq21           GTCTCGTAGAGGGGGGTAGAATTCCAGGTGTAGCGGTGAAATGCGTAGAGATCTGGAGGA
seq33           GTCTtGTAGAGGGGGGTAGAATTCCAGGTGTAGCGGTGAAATGCGTAGAGATCTGGAGGA
seq67           GTCTtGTAGAGGGGGGTAGAATTCCAGGTGTAGCGGTGAAATGCGTAGAGATCTGGAGGA
seq35           GTCTtGTAGAGGGGGGTAGAATTCCAGGTGTAGCGGTGAAATGCGTAGAGATCTGGAGGA
seq52           GTCTtGTAGAGGGGGGTAGAATTCCAGGTGTAGCGGTGAAATGCGTAGAGATCTGGAGGA
seq19           GTCTCGTAGAGGGGGGTAGAATTCCAGGTGTAGCGGTGAAATGCGTAGAGATCTGGAGGA
seq40           GTCTCGTAGAGGGGGGTAGAATTCCAGGTGTAGCGGTGAAATGCGTAGAGATCTGGAGGA
seq43           GTCTCGTAGAGGGGGGTAGAATTCCAGGTGTAGCGGTGAAATGCGTAGAGATCTGGAGGA

seq20           ATACCGGTGGCGAAGGCGGCCCCCTGGACGAAGACTGACGCTCAGGTGCGAAAGCGTGGG
seq21           ATACCGGTGGCGAAGGCGGCCCCCTGGACGAAGACTGACGCTCAGGTGCGAAAGCGTGGG
seq33           ATACCGGTGGCGAAGGCGGCCCCCTGGACaAAGACTGACGCTCAGGTGCGAAAGCGTGGG
seq67           ATACCGGTGGCGAAGGCGGCCCCCTGGACaAAGACTGACGCTCAGGTGCGAAAGCGTGGG
seq35           ATACCGGTGGCGAAGGCGGCCCCCTGGACaAAGACTGACGCTCAGGTGCGAAAGCGTGGG
seq52           ATACCGGTGGCGAAGGCGGCCCCCTGGACaAAGACTGACGCTCAGGTGCGAAAGCGTGGG
seq19           ATACCGGTGGCGAAGGCGGCCCCCTGGACGAAGACTGACGCTCAGGTGCGAAAGCGTGGG
seq40           ATACCGGTGGCGAAGGCGGCCCCCTGGACGAAGACTGACGCTCAGGTGCGAAAGCGTGGG
seq43           ATACCGGTGGCGAAGGCGGCCCCCTGGACGAAGACTGACGCTCAGGTGCGAAAGCGTGGG

seq20           GAGCAAA
seq21           GAGCAAA
seq33           GAGCAAA
seq67           GAGCAAA
seq35           GAGCAAA
seq52           GAGCAAA
seq19           GAGCAAA
seq40           GAGCAAA
seq43           GAGCAAA
```
